# Underreporting of stillbirths in Pakistan: perspectives of the parents, community and healthcare providers

**DOI:** 10.1186/s12884-018-1924-9

**Published:** 2018-07-16

**Authors:** Muhammad Zakria Zakar, Rubeena Zakar, Mudasir Mustafa, Aisha Jalil, Florian Fischer

**Affiliations:** 1 Department of Sociology, Institute of Social and Cultural Studies, University of the Punjab, Lahore, Pakistan; 2 Department of Public Health, Institute of Social and Cultural Studies, University of the Punjab, Lahore, Pakistan; 3 Department of Public Health Medicine, School of Public Health, Bielefeld University, Bielefeld, Germany

**Keywords:** Stillbirths, Pakistan, Gestational health, Cause of death, Underreporting

## Abstract

**Background:**

Pakistan has the highest rate of stillbirths globally. Not much attention has been given so far to exploring the sociocultural factors hindering the reportage of stillbirths and the causes of death. Therefore, the aim of this study was to assess the perspectives of parents, communities and healthcare providers regarding the sociocultural practices and health system-related factors contributing to stillbirths and their underreporting.

**Methods:**

This study used a qualitative approach including in-depth interviews and 14 focus group discussions to collect data from four districts of Pakistan. We conducted 285 in-depth interviews and 14 focus group discussions with health professionals – mainly active in the areas of maternal and child health – and parents who had experienced stillbirth. Constant comparative method and analytical induction method were performed to analyze the data.

**Results:**

The results of this study show that stillbirth is frequently misclassified and, therefore, an underreported phenomenon in Pakistan. It is an outcome of sociocultural practices, such as the social meaning of stillbirth and their understanding about the conflict between cultural and medical anatomy. In addition to grief and psychological distress, it endangers the maternal identity and worth in society in contrast to the mothers of live-born children.

**Conclusion:**

The misclassification of stillbirth, especially by healthcare providers, is a significant impediment to designing preventive strategies for stillbirth. We recommend that the reporting system for stillbirth should be aligned with the WHO definition of stillbirth to avoid its underreporting. Reporting procedures at a more administrative level need to be made uniform and simplified.

## Background

The birth of child with no sign of life, such as breath, voluntary muscle movement and heartbeat, after 28 weeks of pregnancy is known as stillbirth [1, 2]. The main causes of stillbirths are hypertension, eclampsia, abruptio placenta, birth asphyxia, preterm labor, inadequate antenatal care and suboptimal intrapartum [3]. It is important to distinguish among the pregnancies which does not result in a live child to improve the registration of fetal deaths as a vital part of global newborn survival efforts [4]. Global health statistics showed that around 2.6 million stillbirths occur annually, of which 75% takes place in low- and middle-income countries [5, 6]. Pakistan was the country with the highest stillbirth rate (43.1 stillbirths per 1000 total births compared to a global estimate of 18.4) globally in 2015 [7]. Despite the relevance of stillbirths, it remains an unrecognized issue in several countries, including Pakistan.

High stillbirth rates may be attributed to the lack of proper gynecological and obstetric care, poor maternal health, inadequate modes of delivery, violence in gestation and sociocultural practices [8, 9]. Additionally, the risk of stillbirth is high for low- or overweight fetuses [10]. Empirical evidence revealed that 2.6 million stillbirths take place annually in rural areas away from health facilities [11]. Similarly, the high incidence of stillbirth in Pakistan can be attributed to the delayed healthcare-seeking behavior, especially in rural areas due to the lack of accessible and quality healthcare facilities [12].

Research has revealed that stillbirths are not reported to hide the errors in healthcare provision [13]. Effective reporting depends on accurate knowledge regarding stillbirth, specifically how it is distinguishable from neonatal deaths. The records of child mortality and causes of death (COD) are maintained through vital statistics reporting systems in developed countries. However, the COD is mostly not recorded for stillbirths, particularly in developing countries. The vital registration data of Pakistan, for example, did not include COD for approximately 97% of perinatal deaths [14]. In addition, the District Health Information System (DHIS) and Lady Health Workers Management Information System are used to record child health- and mortality-related information in Pakistan. The DHIS report for 2013 lacks any kind of information on stillbirths and related COD [15].

The identification of the contextual and maternal factors leading to potentially preventable stillbirths can help to control the occurrence of stillbirths [16]. Stillbirths may lead to parental depression and sometimes condemnation for mothers by the community [15]. Moreover, a growing body of literature on stillbirths highlighted that statistics about stillbirths are not accurate and lead mostly to an underreporting in developing countries. This is due to various reasons, including the stigma associated with stillbirths [16–18]. Given this backdrop, the aim of this study is to understand sociocultural- and health system-related factors hindering the timely reporting of stillbirths.

## Methods

### Study design and data collection

This study used a qualitative approach including focus group discussions (FGDs) and in-depth interviews (IDIs) to collect data from four districts of Pakistan (Nowshehra, Mardan, Tando Allah Yar and Thatta) from the two provinces of Sindh and Khyber Pakhtunkhawa (KPK). The participants were recruited using purposive and snowball sampling. At initial stage, the researchers contacted with district healthcare officer/manager to get access to district coordinators, and Lady Health Workers (LHW) or Community Midwives (CMWs) and Traditional Birth Attendants (TBAs). The LHWs, CMWs, and TBAs provided further access to couples and women who had experienced stillbirths in past 5 years.

The FGDs were conducted at first and the participants who were found to be more informative were also recruited for IDIs. The participants of FGDs provide referrals to more women and men for IDIs. A total 14 FGD (7 with men and 7 with women) were conducted in both provinces of Pakistan (see Table 1). In each FGD, around 6 to 8 participants were recruited, thus, a total 42 women and 46 men – who had experienced stillbirth – participated in FGDs.

**Table 1:** Participants, sample size and methods of data collection

	Participant categories	Methods of data collection	Number of interviews	Province
1	Health facility in charge, Lady Health Visitor, Medical Technician	In-depth interview	124	Sindh: 62
				KPK: 62
2	Lady Health Worker, Community Midwife	In-depth interview	65	Sindh: 32
				KPK: 33
3	Women who experienced a stillbirth	In-depth interview	50	Sindh: 21
				KPK: 29
4	Traditional Birth Attendants	In-depth interview	36	Sindh: 19
				KPK: 17
5	Coordinators (e.g. for LHW, District Health Information System, Maternal Neonatal Child Health program)	In-depth interview	10	Sindh: 6
				KPK: 4
6	Men and women who experienced stillbirth	Focus group discussion	14	Sindh: 7
				KPK: 7

After FGDs, a total of 285 IDIs were conducted with a variety of stakeholders. Of which, 10 IDIs were conducted with District Health Coordinators, 124 with staff from the Health Facility in Charge, 65 with LHWs or CMWs and TBAs (Table 1). Each IDI was conducted at the desired place of the participants. All district health coordinators and in-charge of health facility, LHWs, CMWs, and TBAs provided interviews at health facility. However, more than half of the participating women also requested to provide the interview at the health facility as they found it to be a comfortable place to talk. Thus, the researchers took more than half of the interview at the health facility, while the rest was taken at the respective home of the women.

Both IDIs and FGDs are conducted in their native or mother language, Sindhi and Pashto. For this, a total of 10 female and two male researchers, who spoke the native language as well as were able to speak and write national and official language of Pakistan, Urdu and English respectively, were hired to conduct the IDIs and FGDs. A two-day training was organized for them. The IDIs and FGDs were recorded after the consent of the participants had been granted. Healthcare providers who had had more than 1 year of work experience and women and men participants who had experienced at least one stillbirth were included in the study.

The study followed ethical issues throughout research. The participation in study was voluntarily and the study participants were told that they can drop at any point of the study. The privacy and confidentiality of the participants were maintained, thus, the name and addresses of the participants were neither asked nor recorded at paper or audio-recorded. In addition, the security concerns of researchers and study participants were also catered and the IDIs and FGDs were conducted at health facility, which were considered safest by study participants. In case, when the study participants were requested to provide interview at their homes, then, the trustworthy LHWs and CMWs accompany the female researcher, while the health-facility in-charge or male dispenser of the health facility accompany the male researchers.

### Data analysis

The data collected through IDIs and FGDs were transcribed into verbatim Urdu (when the conversations were in Sindhi and Pushto [local languages], they were first translated into Urdu with the help of native speakers) and then translated into English. It was ensured throughout the process of translation that the contextual meaning intended by the participant’s statements did not get lost. We used the constant comparative method [19] and analytical induction method of data analysis [20] to generate causal explanations of phenomena. In this regard, we studied a few cases of the phenomenon to be explained initially and looked for similar factors. The explanation for further cases was established after developing a hypothetical explanation based on the primary analysis. The researchers read the translated data thoroughly and moved back and forth in the transcripts. The themes were identified from the data based on the similarities and differences of the views/responses of the participants. Subsequently, various sub-themes were identified under each theme based on the frequency of the codes. The data validity and triangulation of findings were ensured by frequent meetings and analysis by the researchers.

## Results

The findings are presented under four main themes: community views on the nature of pregnancy and stillbirth, women’s experiences of stillbirth reporting, stillbirth causation and the reasons for underreporting. Each theme has more than two sub-themes which are presented as follows.

### Community views on the nature of pregnancy and stillbirth

#### Sociocultural practices

It was found that women’s access to health facilities was restricted due not only to their unavailability in their vicinity, but also to the cultural inhibitions and reliance on male family members (such as a brother, father or husband). Healthcare accessibility is controlled mainly by the male head of the household and mothers-in-law. It is usual cultural practice that the mother-in-law decides about healthcare utilization for her daughter in-law. In most cases, the mother-in-law is not in favor of seeking emergency services, and this may lead to the death of the fetus. Women related that they were not taken to the hospital when a complicated pregnancy arose. Poverty was one reason cited for such undesirable situations, yet some women blamed themselves for the negligence:

*Poverty devastated me; I had four stillbirths in a row. Most people are illiterate and poor here. They are conservative; they do not allow their women to go for check-ups. But rich women do not face such issues; they have more freedom than us.*

A TBA reported similar experiences:

*Women are not allowed to visit the health facility center without being accompanied by a male relative or an older woman of the house. For this reason, I went for irregular antenatal check-ups.*

#### Stoicism and sorcery

Most community members and parents referred to the causes of stillbirths that were rooted in the local religious and cultural context. People believe that stillbirths are an outcome of the evil eye, black magic, curses and *taweez* (an amulet or locket usually containing verses from the Quran or other prayers and symbols) which is inflicted by jealous relatives*.* A feeling of fear and helplessness was observed from the discourse of participants in this regard. A male FGD participant said:

*Taweez and black magic are the reasons for child deaths before birth and often these are performed by other female relatives.*

By contrast, another very consistent response was that the life and death are at the sole discretion of Allah (God) and it was his will that caused the stillbirth:

*You cannot stand before the will of Allah. Each second of our life is predetermined and these cases (stillbirths) are essentially a test by Allah to check our faith and patience.*

In some cases, women were not allowed to carry the babies of other women because the former were ill-fated and, thereby, considered to be untouchables:

*My sister-in-law did not allow me to hold her newborn baby. She said that I killed (lost) my two babies and she did not want to have such misfortune.*

Mothers of stillborn babies were also not allowed to attend celebrations to welcome the unborn baby. Women are not invited to such happy occasions as a reminder of their lost child. However, most participants refuted this proposition and argued that women were not invited simply because they were considered “sinful child-killers.” A woman described such a situation:

*I was not allowed to place fruit in my sister’s lap (a ritual) at her* gaud bharai *(a celebration to congratulate an expecting mother in the seventh month of her pregnancy). I cannot explain the pain of such a rejection by my own family members.*

As a response to social discrimination, some women consulted religious and traditional healers to avoid stillbirth in the future. These healers give the women either *taweez* (amulets) to wear around the neck or some kind of ‘holy’ water to drink.

### Indigenous terminology and understanding about stillbirth

The terminology used for stillbirth varies by sociocultural milieu among the regions in Pakistan. The data analysis demonstrated that the meaning of stillbirth was unclear and vague to the parents, community and healthcare providers. Labelling the reality by a local word may not be the issue, but it becomes a matter of concern by the virtue of connotations attached to it. The parents and families not only had a misplaced understanding of the concept of stillbirth, but the healthcare providers also wrongly defined and, thus, erroneously classified it in a certain category. Most of the time, for example, they were unclear about the difference between miscarriages, stillbirths and neonatal deaths, which resulted consequently in the underreporting of the cases of stillbirth. Hence, stakeholders were asked to clarify the meanings of stillbirth to assess the validity of reporting.

Male and female participants of FGDs exhibited a deficient understanding about stillbirth. Of the 14 FGDs conducted, there were only two such discussions where the participants defined the concept of stillbirth correctly. It is also significant to note that there were no marked differences between the knowledge of male and female FGD participants regarding stillbirths. In addition, the discussion about stillbirths was judged as useless:

*Why are you people wasting time discussing things which are not in our hands? If a fetus dies in the abdomen of mother, who knows the cause? Who knows the time? It is by the will of Allah. He knows better.*

In addition, male and female participants during the FGD used the word *batcha* for a fetus whether it was dead or alive. The place of *batcha* in the uterus was also culturally defined and understood; some mothers thought that the *batcha* is positioned somewhere in the abdomen, while others considered it was in the *bitcha-e-dani* (the lower abdomen/uterus). Keeping such folk anatomy in mind, women were advised to drink hot milk with ghee (butter with the fat removed) so that a slippery situation in the *bitcha-e-dani* facilitates the delivery. Any action of women which deviates from such cultural understanding was considered to be a cause of stillbirth. Thus, we found that a lack of understanding about stillbirth was augmented because of inconsistent terminologies (a wide gap between biomedical and local terminologies).

The entire process of delivery, its method, competence of the care provider, correct temperature maintained at the place of delivery, appropriate method of delivery, handling of mother and child at the time of delivery and afterwards (e.g. massage of the abdomen, position of the mother, type of food given to the mother at the time of delivery) were all comprehensively defined by the local culture with appropriate language and terminology. Additionally, the local culture had a readily available ‘tool-kit’ for all these steps, processes and procedures, which was shared by the local population. The phenomenon of stillbirth was conceived, interpreted and reported with this cultural understanding of the entire reproductive process.

### Women’s experiences of stillbirth reporting

#### Threat to maternal identity

The participants reported the perception that any disability or weakness, such as stillbirth, needs to be kept secret, at least as far as possible. This is because no one wants to be stigmatized with any disability, primarily because of its negative implications regarding one’s image in society. A common belief is that a woman brings her fortune and misfortune with her to her in-laws. If something adverse happens, women are blamed as ill-omened. A woman is threatened with a second marriage of her husband by the in-laws. Therefore, mothers experiencing stillbirths have to face dehumanization, humiliation and sometimes social exclusion. The healthcare system in Pakistan often tends to reveal the identity of patients, though the government has been providing training to healthcare staff (particularly community-based healthcare staff and providers) to maintain the confidentially of the patients. However, the community healthcare workers often reveal the identity of the women who have experienced stillbirths. Therefore, mothers do not report a stillbirth to avoid any associated threats.

#### Avoidance of isolation and shame

The findings revealed that stillbirths are less reported by women to avoid getting blamed and being stigmatized. The LHWs highlighted adverse social consequences for women who experienced stillbirth. They pointed out that in the case of one stillborn baby, some families who were educated and knowledgeable took it as “Allah’s will” and did not blame the woman or anyone else for this happening. In fact, they analyzed the situation resulting in stillbirth and, thereby, started to take care of the mother and followed the appropriate treatment. In cases of repeated stillbirths, women reported becoming dishonored and stigmatized as ominous or unfortunate. In addition, they frequently reported neglect and mistreatment by their husband and his family. In extreme situations in the case of repeated stillbirths, women were even divorced.

It was noted during the male FGDs that women were often blamed for not taking care of their pregnancy. Contrary to the views expressed by the female interviewees, men believed that it was the responsibility of the woman to manage her dietary habits and health issues during pregnancy. Maternity-related issues were considered by some male participants as an exclusively female domain where the women should discuss any problems with other females at home or with female neighbors:

*We (males) remain outside the home for most of the day. Females understand these issues better and they should deal with them at their own level.*

Another male participant provided the following statement:

*Both, family and women are responsible for stillbirth, but it is obvious that the woman is to be blamed more for her carelessness.*

Women who had experienced stillbirth stated that they were not only ostracized by their men but also by other women of the family.

Not a single woman who experienced stillbirth in the sample confirmed that they reported the stillbirth to DHIS or vital registration. The disconnect between government health facilities and private clinics/hospitals for DHIS-based data collection was frequently reported as a cause of lower stillbirth reporting.

### Stillbirth causation

#### Social mobility and healthcare accessibility

The qualitative data analysis revealed that the long distances between the house of the pregnant woman and the nearest health facility, lack of availability of transport, restricted mobility of (pregnant) women, delayed healthcare-seeking behavior and irregular antenatal visits contributed significantly to the occurrence of stillbirths. One TBA, for example, endorsed this issue by stating:

*After feeling labor pains, waiting for a male guardian to come home sometimes extends for hours, which increases the intensity of the complexity of the delivery.*

Several babies were reported to have died in their mothers’ wombs before reaching the nearest health facility. In most of the cases, public health facilities were located far away from the residence of the expectant mothers, which constrained their ability to use the facility. Another woman shared similar experiences:

*My doctor told me that my baby’s position was abnormal, so I had to report to doctor when pains came. Unfortunately, my husband was not at home at that time, so delayed contact to the doctor resulted in the death of my baby.*

The participants frequently mentioned in the FGDs that healthcare accessibility varies between women depending on their financial resources. The socioeconomic dependence on husbands was also found to be linked with pregnancy problems. Women could not spend money on their health during pregnancy, which was related as follows:

*Women cannot spend money on their health. Male members of family say that it is useless to go to the doctor both before and at the delivery.*

It was implicit in the comments of some women that their families or husbands were responsible for not taking care of them:

The doctor advised me to take multivitamins and calcium tablets, but I was not able to go to the pharmacy alone.

*I had been having severe pains since the early morning that day, but my husband came home late from work. I think it was all over by the time he took me to the hospital.*

#### Major deficiencies and diseases

Regarding medical grounds, nutritional deficiency, anemia, and high or low blood pressure were cited as frequent medical problems experienced during pregnancy by the women who had had a stillbirth. Most of the women attributed the cause of the stillbirth to lack of proper food at the proper time. Others considered propriety not in food itself, but also in the process of cooking:

*When I was pregnant, my mother in-law always gave me inappropriate food. My husband said that beef was not good during pregnancy, but my in-laws never cared about this. They frequently cooked beef. Since nothing else was available at home, I used to have beef and you can see its result [referring to the stillbirth*.

The TBAs agreed that anemia, malnutrition, dehydration and high blood pressure were the most common reasons for the occurrence of stillbirths. They used complex local terminology mixed with biomedical concepts while expanding such reasons. Sometimes they tried to converge and connect the meaning of both medical systems by using their own culture-specific knowledge. One TBA, while explaining high blood pressure as the cause of adverse pregnancy outcomes, narrated:

*A child is very tender and vulnerable in the womb. It can die with the pressure and excessive heat. When the mother has blood pressure and she also works in the kitchen for long hours, it creates a double burden and heat on the fetus. If mother is then denied cold and fresh food, the child will die.*

Women also used different terminologies when talking about the causes of stillbirth, and the mother was usually blamed for the baby’s death. It was observed that the women were frequently not informed about the cause of stillbirths by the healthcare professionals:

*The doctor did not tell us (about the cause of stillbirth), neither did we ask. I do not know anything more than that. I lost my child before his birth.*

*I strongly believe that my child was born alive and I could feel movement until the last moment. I do not know why it was a stillbirth.*

#### Mishandling of pregnancy-related complications

Some women stated that the health professionals initially told them that their case was normal, but when they went to the health facility, they were operated on. These women considered that an unwarranted operation was the cause of the stillbirth:

*Half of the child was out and they were trying to pull it. Then it was pushed back and they operated. This was why the baby died.*

It was also reported that there was a deep distrust between the care provider and the mother. One reason for this distrust was that the care providers never took the mother or her relatives into their confidence when making the decisions. A male participant recorded his strong suspicions about the unnecessary medical procedures recommended or even carried out by care providers in order to make money:

*Doctors have stories to tell each patient. Firstly, they create fear by saying that there is not enough fluid, the baby’s movement is low or the cord is around the neck. Then they start preparing for a major operation (referring to caesarean section). They just fool people. This was how they killed my brother’s unborn son.*

### Reasons for underreporting

#### Understanding of healthcare providers about stillbirth

The findings showed that about two-thirds of the LHWs were unable to differentiate clearly between stillbirths and other adverse pregnancy outcomes and neonatal deaths. It may be assumed that they would not be reporting stillbirth cases accurately. One LHW with several years of experience as a community care provider said:

*Stillbirth refers to either the death of the child within one to one days after birth or the death of the child in the mother’s womb*.

Although, according to this definition, she considered perinatal death as a stillbirth, it was interesting to note that she had not observed a stillbirth in her area. Since management information systems rely on the reporting of Lady Health Workers Management Information Systems for the collection of primary data, the restricted ability of any LHW to differentiate between various kinds of child mortalities obscures the credibility of the whole process of the information systems in place. Another LHW stated her confusion about the reporting of stillbirths:

*It is very confusing to define stillbirth. I have asked the Lady Health Supervisor about the difference between a stillbirth and a neonatal death. She said, it’s almost the same, so we can report it any way we want.*

It was also noted that some LHWs did not consider it necessary to understand a clear distinction between miscarriage, fetal death and stillbirth. Like the general public, they used local terminology to explain the reality; the problem with the local terminology was that it was very vague and embedded in the cultural anatomy, which is the cultural understanding of the body. However, this cultural anatomy may not correspond to the biomedical understanding of the human body and its functioning. For example, in the local culture, Stillbirth, for example, is understood in the local culture as a fetus who has died in the *pait* (means literally “abdomen”) of the mother. Technically, the fetus is not positioned in the abdomen but in the uterus. When health care providers, especially non-doctors, use local terminology and subscribe to the local world view, it distorts the accurate information.

#### Mistrust of healthcare providers

Poverty, sociocultural practices, lack of adequate services/staff availability at government facilities and transport problems were the reasons for using the services of TBAs as birth attendants. The magnitude of relying on the TBAs can be assessed by the claim of one TBA who reported having conducted 3000 deliveries in 11 years. Since the role of TBAs has a cultural connotation in many villages, it was noted that some interviewees showed of mistrust of healthcare providers and were more confident with the proficiency of TBAs – such as Dais – than skilled health care providers:

*The Dai in our village is very experienced. If she could not save my baby, it was Allah’s will. There is no need to report it to anyone*.

#### Missing capacity and competency for reporting

The capacity and competency of the medical officers and other administrational support staff for handling and maintaining the health information system’s data including the stillbirths was a matter of serious concern. It was reported that most of the staff members, such as medical technicians, dispensers, nurses and even medical officers, were not adequately trained to handle different formats of registers and reports for retrieving and compiling information on stillbirths. The task was delegated to one or two staff members who had some relevant competency for recording and retrieving data from different sources and compiling it for preparing monthly reports for timely submission to the district management. The absence of systematic mechanisms for monitoring and quality assurance was claimed; staff members themselves were responsible for ensuring the accuracy of the information. In the case of their leaving or being absent from duty, the data was not maintained and updated by other staff members due to a lack of relevant expertise.

#### Lack of institutionalized coordination among healthcare providers

Healthcare providers at the community level include LHWs, TBAs and CMWs. The LHWs have the primary role of collecting and recording stillbirth-related information. Additionally, TBAs and CMWs contribute by reporting stillbirths under the maternal, newborn and child health (MNCH) program. Data gathered from IDIs with LHWs, CMWs and TBAs revealed that all these functionaries belonged to the same local communities where they worked. Most of them knew one another and shared information on mother and child health-related issues routinely, including the cases of stillbirths. However, they did not have any institutionalized and structured mechanism for sharing such information. They could only receive such information as a matter of chance during their occasional interactions while moving around in the community.

Some of the LHWs opined that collecting and reporting information on different registers, cards and reports was a merely futile exercise, because they did not see that these reports could be of any use to higher authorities. They were also of the view that nobody bothered to check the reports and act accordingly; it was mere waste of time and money. They were convinced that information and monthly reports could only be of importance if the government started programs for controlling stillbirths. This was accentuated by one LHW as follows:

*Preparing monthly reports on stillbirths is merely a formality and nothing more than that. Nobody is interested why this is happening. When the government does not do anything to control stillbirths, then why we are pressurized to submit reports every month?*

#### Perceived uselessness of reporting

The TBAs included in the study were asked to give insights into the community trends about reporting stillbirths. Almost two-thirds of the TBAs agreed that the women or members of their households did not share information about stillbirths:

*If the stillbirth occurred at a private hospital or through home delivery, then the families did not bother to inform us and considered it unnecessary*.

TBAs thought that people considered reporting unnecessary because it could not bring their babies back. Some TBAs were themselves critical of the usefulness of reporting:

*When nobody, neither managers nor families, care to take or adopt any preventive measures to avoid stillbirths or adverse pregnancy outcomes, then it is a useless activity*.

In addition, the community perception about the lower prevalence of stillbirths undermines the reporting of stillbirth cases. The outcomes of the FGDs revealed that the prevalence of stillbirth was considered to be very rare.

## Discussion

The data analysis demonstrated that the reporting behavior of stillbirths was associated with stigma [11, 21]. Another significant factor was the inaccurate understanding about the meaning of stillbirth by both healthcare providers and the community [22]. Previous research has already shown that mothers were frequently not sure whether their child was stillborn and what caused the death of their child [14]. We noted restrictions on women mobility and their dependency on males regarding the utilization of health services was a major factor contributing to poor pregnancy outcomes such as stillbirths. Our findings are aligned with research previously conducted in other developing countries including Pakistan [23, 24].

In addition, the local terminologies used for stillbirth in the four districts selected were inconsistent, vague and indicated misperceptions about its definition and causes. Women were stigmatized and blamed for delivering a stillborn child in all four districts. Women experiencing repeated stillbirths felt discriminated against and socially excluded by the community. In addition to maltreatment by their husbands, the other female members of the family also ostracized the women who experienced stillbirths. Consequently, women had low self-esteem and a sense of dejection, which may increase the likelihood of further adverse health outcomes [25].

There was absence of acceptance of caesarean section and women did not realize that the occurrence of stillbirths can be reduced through the provision of emergency obstetric services. By contrast, many healthcare providers believed that stillbirths cannot be prevented [26]. Moreover, the fatalistic attitude of the parents contributes to the number of stillbirths, because many people believe that “the baby was not destined to live.” We also found that the stillbirths leave psychosocial impacts on mothers who have experienced them, which has already been extensively studied and proven empirically [27]. It is considered the mother’s fault due to inappropriate diet or lifestyle, and a consequence of their sins/evil spirits [9, 16].

This study fills the gaps in the literature by providing an understanding of health providers, male community members and women who have experienced stillbirth. Additionally, this study helps towards understanding the local context and circumstances influencing stillbirth and its reporting in Pakistan. Collection of data from all tiers of stakeholders and community members is the major strength of this study. The study is well triangulated by using multiple methods of data collection. However, this study has certain limitations. The scope of the study did not include the voice of the older women of the family who have a major say in decision-making regarding seeking medical care in pregnancy. Information on health-related issues was based on self-reports of women and were not clinically proven. Recall bias and socially desirable answers by the participants were some further limitations.

## Conclusion

Comprehensive strategies are needed to improve community participation and understanding about stillbirth. The reporting system for stillbirths should be aligned with the WHO definition of stillbirth [28]. Early screening and identification of high-risk pregnancies can prevent stillbirths. Specialized training and refresher courses are needed for LHWs, CMWs and other healthcare providers to screen and refer the cases of high-risk pregnancies to the health facilities. There is a need for raising the awareness and knowledge of mothers and families about the causes and risk factors leading to stillbirths with scientific evidence [29]. Community awareness and advocacy programs for better maternal health should be organized by health departments through designing media-based customized information programs regarding MNCH, and displaying awareness posters at health facilities,

Since the attitude and behavior of men are one of the important factors, it is imperative to improve the health literacy of the communities by involving them, particularly local religious leaders and men in MNCH awareness programs. Cultural narratives about stillbirth and stigmatization of women who experience stillbirth are a major bottleneck in implementing effective responses against the issue [30]. It is important to challenge the societal stereotypes about stillbirth by enhancing the communities’ awareness and improving health literacy about the causes of stillbirth and the significance of its reporting.

## Abbreviations

CMW: Community Midwife
COD: Cause of death
DHIS: District Health Information System
FGD: Focus group discussion
IDI: In-depth interview
KPK: Khyber Pakhtunkhawa
LHW: Lady Health Worker
MNCH: Maternal neonatal child health
TBA: Traditional Birth Attendant
WHO: World Health Organization
